# Home‐Based Intervention to Test and Start (HITS): a community‐randomized controlled trial to increase HIV testing uptake among men in rural South Africa

**DOI:** 10.1002/jia2.25665

**Published:** 2021-02-15

**Authors:** Frank C Tanser, Hae‐Young Kim, Thulile Mathenjwa, Maryam Shahmanesh, Janet Seeley, Phillippa Matthews, Sally Wyke, Nuala McGrath, Oluwafemi Adeagbo, Benn Sartorius, Handurugamage Manisha Yapa, Thembelihle Zuma, Anya Zeitlin, Ann Blandford, Adrian Dobra, Till Bärnighausen

**Affiliations:** ^1^ Africa Health Research Institute Durban South Africa; ^2^ Lincoln International Institute for Rural Health University of Lincoln Lincoln United Kingdom; ^3^ School of Nursing and Public Health University of KwaZulu‐Natal Durban South Africa; ^4^ Centre for the AIDS Programme of Research in South Africa (CAPRISA) University of KwaZulu‐Natal Durban South Africa; ^5^ Department of Population Health New York University School of Medicine New York NY USA; ^6^ KwaZulu‐Natal Innovation and Sequencing Platform KwaZulu‐Natal South Africa; ^7^ Institute for Global Health University College London London United Kingdom; ^8^ London School of Hygiene and Tropical Medicine London United Kingdom; ^9^ University of Glasgow Glasgow United Kingdom; ^10^ University of Southampton Southampton United Kingdom; ^11^ Department of Sociology University of Johannesburg Johannesburg South Africa; ^12^ Department of Health Promotion, Education and Behaviour University of South Carolina Columbia SC USA; ^13^ The Kirby Institute University of New South Wales Sydney NSW Australia; ^14^ University College London Interaction Centre University College London London United Kingdom; ^15^ University of Washington Seattle WA USA; ^16^ Heidelberg Institute of Global Health University of Heidelberg Heidelberg Germany

**Keywords:** Home‐based HIV testing, men, financial incentives, counselling, mHealth, randomized controlled trial, South Africa

## Abstract

**Introduction:**

The uptake of HIV testing and linkage to care remains low among men, contributing to high HIV incidence in women in South Africa. We conducted the “Home‐Based Intervention to Test and Start” (HITS) in a 2x2 factorial cluster randomized controlled trial in one of the World’s largest ongoing HIV cohorts in rural South Africa aimed at enhancing both intrinsic and extrinsic motivations for HIV testing.

**Methods:**

Between February and December 2018, in the uMkhanyakude district of KwaZulu‐Natal, we randomly assigned 45 communities (clusters) (n = 13,838 residents) to one of the four arms: (i) financial incentives for home‐based HIV testing and linkage to care (R50 [$3] food voucher each); (ii) male‐targeted HIV‐specific decision support application, called EPIC‐HIV; (iii) both financial incentives and male‐targeted HIV‐specific decision support application and (iv) standard of care (SoC). EPIC‐HIV was developed to encourage and serve as an intrinsic motivator for HIV testing and linkage to care, and individually offered to men via a tablet device. Financial incentives were offered to both men and women. Here we report the effect of the interventions on uptake of home‐based HIV testing among men. Intention‐to‐treat (ITT) analysis was performed using modified Poisson regression with adjustment for clustering of standard errors at the cluster levels.

**Results:**

Among all 13,838 men ≥ 15 years living in the 45 communities, the overall population coverage during a single round of home‐based HIV testing was 20.7%. The uptake of HIV testing was 27.5% (683/2481) in the financial incentives arm, 17.1% (433/2534) in the EPIC‐HIV arm, 26.8% (568/2120) in the arm receiving both interventions and 17.8% in the SoC arm. The probability of HIV testing increased substantially by 55% in the financial incentives arm (risk ratio (RR)=1.55, 95% CI: 1.31 to 1.82, p < 0.001) and 51% in the arm receiving both interventions (RR = 1.51, 95% CI: 1.21 to 1.87 p < 0.001), compared to men in the SoC arm. The probability of HIV testing did not significantly differ in the EPIC‐HIV arm (RR = 0.96, 95% CI: 0.76 to 1.20, p = 0.70).

**Conclusions:**

The provision of a small financial incentive acted as a powerful extrinsic motivator substantially increasing the uptake of home‐based HIV testing among men in rural South Africa. In contrast, the counselling and testing application which was designed to encourage and serve as an intrinsic motivator to test for HIV did not increase the uptake of home‐based testing.

## 1 INTRODUCTION

Despite the successes and implementation of population‐wide HIV prevention and treatment programmes [1, 2], female adolescents and young women have consistently faced a high risk of HIV infection, whereas HIV incidence steadily decreased among males, widening the gender gap in many generalized HIV epidemic settings [3, 4, 5, 6]. Men are less likely to test for HIV and link to care once tested positive, leading to worse long‐term health outcomes such as unsuppressed viral load and higher HIV‐related mortality among males and reciprocally high HIV incidence among females [1, 7]. In rural South Africa, once antiretroviral therapy (ART) coverage among males surpassed 35% in 2014, population‐level HIV incidence among females significantly declined [8].

Recent community‐based trials of universal testing and treatment (UTT) including our Test‐as‐Prevention (TasP) trial (ANRS 12249) in rural South Africa achieved no significant or somewhat modest reduction in HIV incidence at the population level [9, 10, 11, 12]. One of the key challenges consistent across these trials was missing men and young people – who were disproportionally reached and consented for HIV testing, and less likely to link to and remain in care [12]. The trials highlighted that it is not sufficient to merely provide home‐based HIV counselling and testing and universal treatment, but effective strategies must be accompanied to increase the uptake of HIV counselling and testing (HCT) and linkage to care at clinics.

Financial incentives have been used to promote various healthy behaviours such as medication adherence [13], reduction of substance misuse [14], or use of preventive services including voluntary medical male circumcision [15, 16, 17]. Recently, several clinical trials showed promising results that financial incentives could increase the uptake of HIV testing as well as linkage to care and viral load suppression but the efficacy has been varying [18, 19, 20, 21, 22, 23, 24, 25, 26]. While financial incentives may serve as once‐off extrinsic motivation, providing information specific to individuals’ needs can improve intrinsic motivation and empower individuals to make an informed decision to take HCT [27, 28].

To our knowledge, no study has explicitly quantified the effect of extrinsic vs. intrinsic motivators on the uptake of HIV testing. We conducted a factorial cluster randomized clinical trial – Home‐based Intervention to Test and Start (HITS) – in rural KwaZulu‐Natal. This trial was built upon the Africa Health Research Institute (AHRI)’s ongoing population‐based HIV testing platform. Since 2017, AHRI started offering home‐based rapid HIV testing to all eligible individuals during annual HIV surveys. The uptake of home‐based rapid HIV testing in 2017 was 26.4% in the overall population and 20.0% among men [29]. The overall aim of the trial is to establish whether the provision of small once‐off financial incentives and a male‐targeted HIV‐specific decision support application increases uptake of HIV testing and linkage to HIV care, thus ultimately reducing population‐level HIV incidence in (particularly young) women. Here we report the uptake of home‐based HIV testing among men, the first registered primary endpoint of the HITS trial.

## 2 METHODS

### 2.1 Setting

The trial was nested within the population‐based HIV testing platform in uMkhanyakude district of northern KwaZulu Natal [30]. HIV prevalence in the study area was estimated as 36.6% in 2016 [31]. Since 2003, AHRI has conducted ongoing population‐based demographic surveillance and individual HIV testing among members of all households residing in the surveillance area. The demographic surveillance is completed with the key informant, which is often the household head or the most senior household member and collects information on demographics of household members including composition, migration events, mortality and marital status. The HIV surveillance is annually conducted among all residents aged ≥ 15 years and collects data on sexual behavior and general health and dried blood spots (DBS) samples for anonymized HIV testing after obtaining informed consent [30, 32]. No reimbursement is offered for participation in the HIV surveillance. From 2017, rapid HIV testing has been offered as part of the HIV surveillance during household visits. The surveillance covers 438 km^2^ geographic area over approximately 90,000 individuals and 60 000 residents at any given time. Through an agreement with the South African Department of Health, the AHRI population‐based programme is also linked with the clinical records of patients enrolled in the local public HIV Treatment and Care Programme at the Hlabisa district hospital and 17 primary health care clinics in the Hlabisa health sub‐district using the Tier.Net electronic record system since 2004 [33]. For the patients who out‐migrate or link to care outside the 17 clinics, their clinical records could not be captured.

### 2.2 Trial design

**Figure 1:**
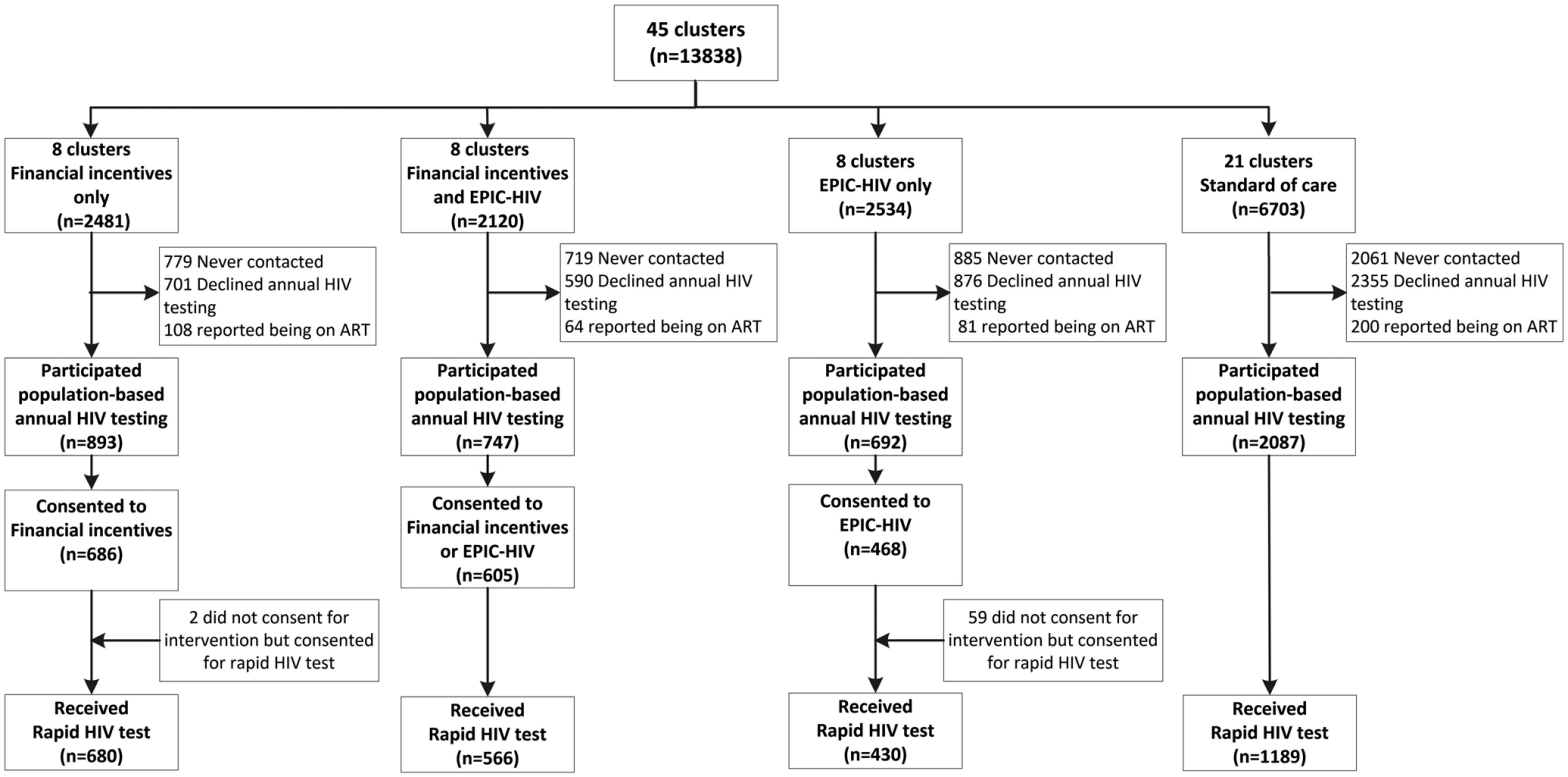
Flow diagram for HITS trial. Flow diagram shows individual flow through each stage of the cluster randomized controlled trial by intervention arms. EPIC, Empowering People through Informed Choices for HIV.

This large‐scale trial was a 2x2 factorial design delivered to a population of 37,028 residents aged ≥ 15 years in the four strata with the two interventions, financial micro‐incentives and a male‐targeted HIV‐specific decision support, called EPIC‐HIV (Empowering People through Informed Choices for HIV), in 45 clusters (“week blocks”) using the AHRI’s ongoing population‐based annual HIV testing platform (Figure 1) [34]. A week block is an area where fieldworkers visit homesteads and complete annual surveys in each week; this was developed using a GPS‐based methodology to provide an equivalent workload to cover the entire surveillance area annually [35]. Over the entire study duration, eight communities received only the financial micro‐incentives (i.e. financial incentives only arm), eight communities received only EPIC‐HIV (i.e. EPIC‐HIV only arm), eight communities received both interventions (i.e. financial incentives plus EPIC‐HIV arm or combined arm) and 21 communities received standard of care (SoC). Both males and females were eligible to receive the financial incentives but only males were eligible to receive EPIC‐HIV. Implementation and acceptance of the HITS intervention were evaluated using process evaluation through post‐intervention satisfaction surveys as well as focus group and in‐depth interviews among study participants, fieldworkers and health professionals. The trial was registered at the National Institute of Health (ClinicalTrials.gov # NCT03757104), and the full trial protocol was published elsewhere [34].

### 2.3 Home‐based HIV counselling & testing and facilitated linkage to care

As part of the individual HIV surveillance, a rapid point‐of‐care HIV test is performed by field workers trained in HCT in accordance with the South African national guidelines. Participants found to be HIV‐positive are encouraged to link to care within seven to ten days of the HIV test date, receive a referral slip for an appointment to receive HIV care at their choice of the primary health clinics, and can opt in for facilitated linkage. Individuals who consent for facilitated linkage and have not linked to care within two weeks of the HIV test date receive a single Short Message Service (SMS) message as a reminder. If individuals do not link to care within further two weeks, a trained nurse contacts them by telephone to discuss any concerns and encourage them to link to care. Individuals who were not available at the initial attempt of contact to complete the HIV surveillance are revisited two more times in the same week during normal working hours and then transferred to a tracking team who then attempts to contact participants and make a home visit for three more times in the evenings or weekends before being considered as missing.

### 2.4 Interventions

The interventions were delivered in a two‐stage scheme for HIV testing and linkage to care. For the financial incentives, participants were first informed and offered a R50 (US ~$3) food voucher for a local supermarket conditional on their participation in rapid HIV testing. Second, participants who tested HIV‐positive were offered another R50 food voucher if they visited any of the 17 primary health clinics to seek HIV treatment within six weeks of the positive HIV test date.

The male‐targeted HIV‐specific decision support application was implemented and offered via a tablet and available in two versions (EPIC‐HIV 1 and EPIC‐HIV 2). The content and design of EPIC‐HIV were informed by human behaviour change theory and self‐determination theory [36]. The application development is described in detail elsewhere [34]. EPIC‐HIV 1 was delivered to men prior to the HIV test offer to support their decision whether to take a rapid HIV test or not. The app was designed to be administered in 5 to 10 minutes. Participants were able to privately self‐navigate the application using a provided earphone and listen to the story of a chosen character. If participants did not link to HIV care within a month of a positive HIV test, a study tracker re‐visited and offered them another counselling application (EPIC‐HIV 2), which is designed to address barriers to seek HIV treatment and encourage them to link to HIV care.

### 2.5 Participants

Individuals were eligible if they were aged ≥ 15 years and residents of the households within the study area in the 45 clusters, agree to participate in the annual HIV surveillance, and willing to give written informed consent for trial participation. At randomization, we estimated that of approximately 37,000 eligible participants in the 45 clusters, a total of 4667 individuals would receive HITS interventions in the 24 intervention clusters and 4900 individuals the standard‐of‐care in the 21 clusters, considering the expected contact rates and participation rates in the annual population‐based HIV testing [34]. Written informed consent was sought for trial participation from all eligible individuals ≥ 18 years, whereas parental/guardian consent with child assent was sought for eligible individuals aged 15 to 17 years.

Individuals were excluded from participation in the study if they have refused to participate in the annual population‐based HIV testing or report to be already on ART. Individuals who self‐reported as HIV‐positive but not on ART were eligible to participate.

### 2.6 Outcomes

We report the uptake of home‐based HIV testing among men, the first registered primary endpoint of the HITS trial. Uptake was defined as acceptance and completion of home‐based HIV testing (with the standard pre‐ and post‐test HIV counselling).

### 2.7 Randomization and blinding

Randomization was conducted to ensure balance across the arms using stratified sampling at the community‐level based on the HIV incidence among young females aged 15 to 30 years. The 45‐week blocks (i.e. community clusters) were divided into four strata according to their HIV incidence rates such that stratum 1 comprised community clusters with the lowest incidence rate, whereas stratum 4 comprised community clusters with the highest incidence rate. Each of the intervention arms consisted of two community clusters from each of the four incidence strata (thus a total of eight community clusters per arm). The SoC arm consisted of five community clusters from each of the incidence stratum 1, 2 and 4, and six community clusters from incidence stratum 3 (thus a total of 21 community clusters). The trial was an open‐labelled without blinding neither to investigators nor participants.

### 2.8 Sample size and power calculation

The study was powered to achieve a 25% reduction in HIV incidence among females aged 15 to 30 years in the intervention arms. Using the AHRI’s actual HIV incidence data, we estimated to be able to detect a 25% reduction in HIV incidence in the financial incentives only arm, 25% reduction in the EPIC‐HIV only arm and a 37% reduction in the combined arm after three years of follow‐up in > 80% of the power of simulation replicates (p < 0.05), accounting for grouping of the clusters in the four arms. Therefore, if we were to introduce the HITS intervention in 2018 and follow young females for at least three years post‐intervention (i.e. utilize a total of 17 years of incidence from 2004 to 2021), we would expect to be in excess of 90% power to detect such a reduction in incidence among females aged 15 to 30 years.

### 2.9 Implementation

The trial statistician (AD) performed the stratified randomization of communities prior to intervention rollout. Fieldwork teams enrolled and offered interventions to participants when eligible. Individual consent was sought after cluster randomization.

### 2.10 Statistical methods

The primary analysis was conducted using the intent‐to‐treat (ITT) analysis for all men randomized at the community level. We used generalized linear models with Poisson distribution and robust error terms, adjusting for community‐level clustering through random effects. We also examined the effect of financial incentives and EPIC‐HIV separately in the factorial analysis and interaction effects between the two interventions. We then limited the analysis to those who participated in the population‐based annual HIV survey and were offered the interventions. All analyses were conducted in STATA 15.1 (StataCorp) and R 3.5.2. The supporting CONSORT checklist and flowchart are available as supplemental information (Figure S1 CONSORT Checklist; Text S1 CONSORT Flowchart).

### 2.11 Ethics statement

The study protocols for the AHRI’s population‐based HIV testing platform and HITS intervention were approved by the Biomedical Research Ethics Committee of the University of KwaZulu‐Natal (BE290/16 and BFC398/16) [34]. Permission for the trial was obtained from the KwaZulu‐Natal Department of Health, South Africa. Participation in the HIV surveillance and HITS trial is completely voluntary. Individuals may choose to opt out or refuse to answer any component of the HIV surveillance and to withdraw at any time. Written informed consent was sought from individuals aged ≥ 18 years, and parental or guardian consent with child assent for individuals of 15 to 17 years old were obtained.

## 3 RESULTS

Overall, all 15,488 men living in the 45 clusters in the study area were initially considered eligible for inclusion in the first primary outcome of the trial – uptake of testing among men. Study participants were enrolled from February 2018 to December 2018. Of these, 1650 died or out‐migrated. Of the 13,838 remaining eligible men, 4444 (32.1%) were never contacted (mainly due to absence at the time of HIV testing despite several attempts to follow‐up), 4522 (32.7%) chose not to participate in the annual population‐based HIV testing in 2018, and 453 (3.3%) reported being on ART, thus resulting in 4419 (31.9% of the resident population) who participated in the population‐based HIV testing (Figure 1). Randomization successfully achieved balance in respect of HIV prevalence and sociodemographic variables across the four arms except for the area of residency (Table 1). Those who participated in the population‐based HIV testing were significantly younger, compared to those who were never contacted or chose not to participate (Table S1).

**Table 1:** Characteristics of communities and eligible male participants by intervention arms in KwaZulu‐Natal, South Africa, 2018

Characteristic	Financial incentives only	Financial incentives plus EPIC‐HIV	EPIC‐HIV only	Standard of care
HIV prevalence in 2018, % (95% CI)^a^	18.5 (15.9 to 21.3)	15.0 (12.4 to 17.9)	19.3 (16.2 to 22.7)	17.6 (15.9 to 19.5)
Time since last HIV test in the surveillance (years), median (IQR)	2.5 (0.6, 6.0)	2.3 (1.0, 5.3)	2.3 (1.0, 5.6)	2.3 (1.0, 5.5)

Individual‐level factors	n = 2481	n = 2120	n = 2534	n = 6703
Age (years), % (n)				
15 to 25	36.5 (906)	37.6 (796)	35.6 (902)	37.2 (2492)
25 to 35	22.5 (558)	23.4 (495)	22.3 (566)	23.4 (1566)
35 to 45	16.5 (410)	13.9 (295)	15.8 (400)	15.7 (1051)
45 to 55	10.4 (258)	10.2 (217)	10.1 (257)	9.7 (647)
≥55	14.1 (349)	14.9 (315)	16.1 (409)	14.1 (946)
Marital status, % (n)				
Never married	26.9 (668)	25.9 (550)	26.2 (664)	26.8 (1798)
Married	14.2 (352)	14.6 (309)	15.6 (396)	14.2 (951)
Informal Union	40.1 (995)	38.3 (811)	37.9 (960)	38.5 (2584)
Separated/Divorced/Widowed	1.4 (35)	2.1 (45)	2.1 (52)	1.9 (125)
Don't know/Missing	17.4 (431)	19.1 (405)	18.2 (462)	18.6 (1245)
Education, % (n)				
No formal education	23.9 (594)	26.0 (552)	25.3 (640)	24.8 (1665)
Primary (grade 1 to 7)	6.3 (156)	6.5 (138)	6.8 (172)	6.4 (432)
Secondary+ (≥ grade 8)	69.8 (1731)	67.5 (1430)	68.0 (1722)	68.7 (4606)
Area of residency, % (n)				
Rural	59.9 (1485)	52.7 (1117)	60.5 (1532)	58.3 (3905)
Peri‐urban	32.8 (813)	47.3 (1003)	26.5 (671)	33.9 (2275)
Urban	7.4 (183)	0.0 (0)	13.1 (331)	7.8 (523)

EPIC, Empowering People through Informed Choices for HIV; IQR, interquartile range

^a^ 95% confidence intervals were calculated using the cumulative probabilities of the binomial distribution.

**Figure 2:**
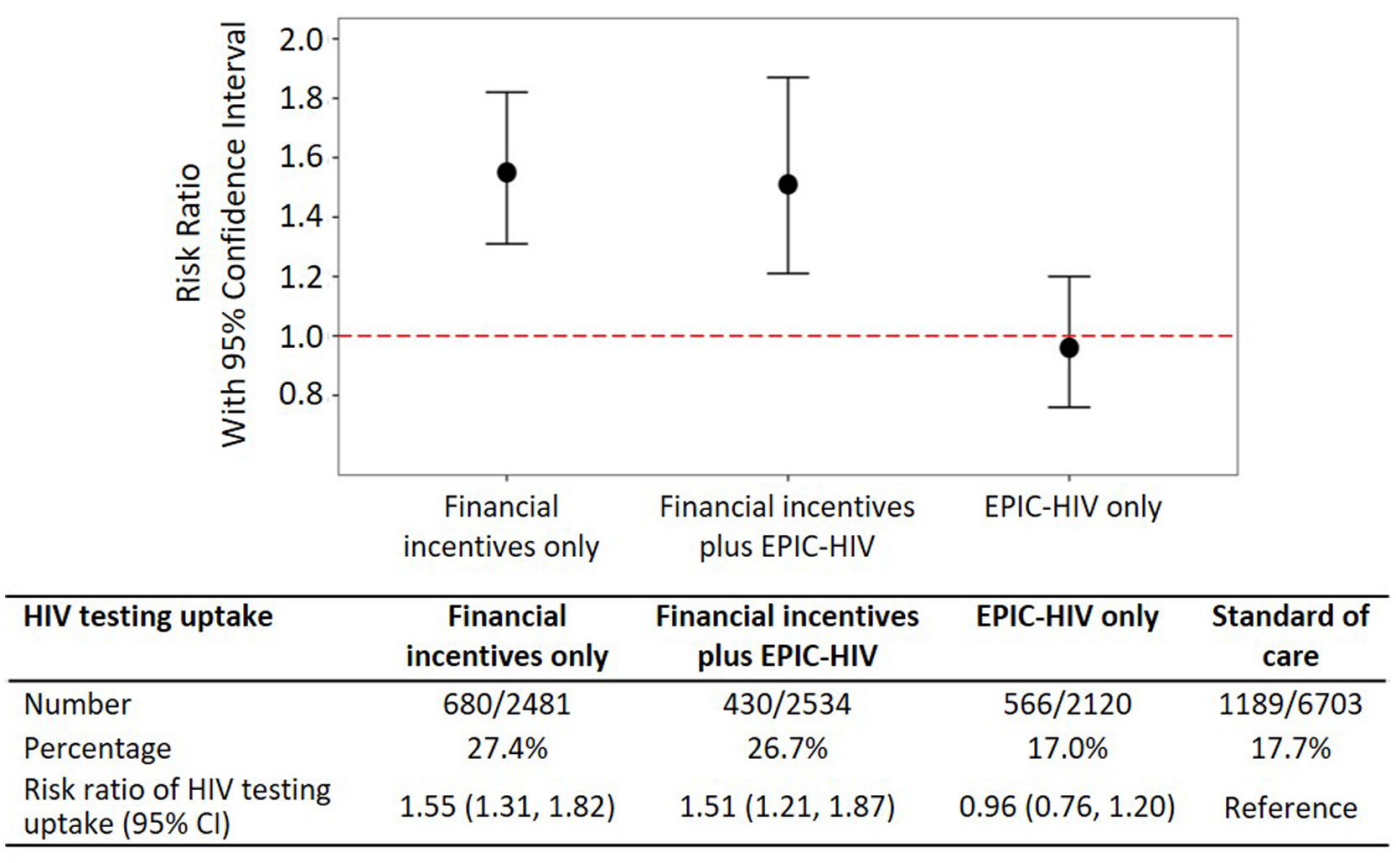
Risk ratio (RR) of HIV testing uptake in the full intent‐to‐treat analysis among all eligible men. The circle symbol with the error bar represents the RR with 95% confidence interval in each arm compared to the standard of care arm. The percentage (%) indicates the percentage of HIV testing uptake.

Among all 13,838 men ≥ 15 years living in the 45 communities in 2018, overall testing coverage was 27.4% (680/2481) in the financial incentives only arm, 17.0% (430/2534) in the EPIC‐HIV only arm, 26.7% (566/2120) in the financial incentives plus EPIC‐HIV arm and 17.7% (1189/6703) in the SoC arm. In ITT analysis, compared to men in the SoC arm, the probability of HIV testing was 55% higher in the financial incentives only arm (risk ratio (RR)=1.55, 95% CI: 1.31 to 1.82, p < 0.001) and 51% higher in the financial incentives plus EPIC‐HIV arm receiving both interventions (RR = 1.51, 95% CI: 1.21 to 1.87, p < 0.001). The probability of HIV testing was not significantly different in the EPIC‐HIV only arm (RR = 0.96, 95% CI: 0.76 to 1.20, p = 0.70) (Table 2; Figure 2).

**Table 2:** Consenting and uptake of HIV testing among all 13,838 eligible men by intervention arms

	Total number of eligible participants	Consented for interventions	Received rapid HIV testing	Risk ratios (95% CI)
	N = 13,838	N = 1759	N = 2865	
		No. (%)	No. (%)	
Intervention group analysis				
Financial incentives only	2481	686 (27.7)	680 (27.4)	1.55 (1.31 to 1.82)
Financial incentives plus EPIC‐HIV	2120	605 (28.5)	566 (26.7)	1.51 (1.21 to 1.87)
EPIC‐HIV only	2534	468 (18.5)	430 (17.0)	0.96 (0.76 to 1.20)
Standard of care	6703	n/a	1189 (17.7)	Reference
Factorial analysis				
Financial incentives	4601	1252 (27.2)	1246 (27.1)	1.55 (1.34 to 1.79)
No financial incentives	9237	n/a	1619 (17.5)	Reference
Factorial analysis				
EPIC‐HIV	4654	1037 (22.3)	996 (21.4)	1.05 (0.85 to 1.30)
No EPIC‐HIV	9184	n/a	1869 (20.4)	Reference

EPIC, Empowering People through Informed Choices for HIV.

When we examined HIV testing uptake by each of the interventions, the provision of financial incentives increased the uptake of home‐based HIV testing by 55% (RR = 1.55, 95% CI: 1.34 to 1.79). EPIC‐HIV did not have any effect on the uptake of HIV testing at the time of testing offer (RR = 1.05, 95% CI: 0.85 to 1.30, p = 0.64). There was no interaction between financial incentives and EPIC‐HIV (p‐value = 0.91).

When we limited the analysis to those who participated in the ongoing population‐based HIV testing and thus were offered the interventions, 76.2% (680/893) in the financial incentives only arm, 75.8% (566/747) in the combined arm, 62.1% (430/692) in the EPIC‐HIV only arm and 57.0% (1189/2087) in the SoC arm consented and received the rapid HIV testing (Figure 1). Compared to men in the SoC arm, the probability of HIV testing was 34% higher in the financial incentives only arm (RR = 1.34, 95% CI: 1.24 to 1.45, p < 0.001) and 33% higher in the combined arm receiving both interventions (RR = 1.33, 95% CI: 1.20 to 1.48, p < 0.001). The probability of HIV testing was not significantly different in the EPIC‐HIV only arm (RR = 1.09, 95% CI: 0.98 to 1.21, p = 0.10).

## 4 DISCUSSION

In this study, we found that a small non‐cash financial incentive (US$ 3) designed to provide an extrinsic motivation to test substantially increased the uptake of home‐based HIV testing by more than 50% when offered either alone or in combination with a male‐targeted HIV‐specific decision support application. In contrast, the decision support application designed to act as an intrinsic motivator did not improve the uptake of home‐based HIV testing at the time of testing offer. Our study is the first large trial to test the effects of extrinsic versus intrinsic motivators on uptake of home‐based HIV testing specifically tailored and targeted to men.

A few studies have shown the positive effect of financial incentives on the uptake of HCT. In recent randomized controlled trials conducted in sub‐Saharan Africa, financial incentives (i.e. both non‐cash and cash incentives) significantly increased the uptake of HIV testing among men in Malawi [19] or adolescents in Zimbabwe [20]. On the other hand, most of the previous cash transfer programmes among women and girls, which focused on changing sexual behaviours through creating positive social protection, found non‐significant or marginal effects [37]. Our results provide strong evidence that the relatively small once‐off micro‐incentives can play an important role in overcoming a barrier for accepting HIV testing at a home environment among men.

There are several explanations as to why financial incentives were well accepted and significantly increased the uptake of HIV testing among men in this trial. We provided a fixed financial incentive which offered immediate, tangible and easy‐to‐use benefit to everyone in the assigned intervention arms, rather than in some delayed manner or a chance of receiving it (i.e. lottery). In Malawi, while fixed incentives improved the uptake of HIV self‐testing kits among men by up to 34%, the chance of winning a lottery for a larger amount did not result in any significant difference [19]. Similarly, when either fixed incentives or a lottery were provided among adolescents in Zimbabwe, fixed incentives better increased HIV testing uptake than a lottery [20]. We then offered home‐based HIV testing which provided the convenient option once someone is nudged to accept testing.

We have not observed any effect of the male‐sensitive HIV‐specific decision support application (EPIC‐HIV 1) on the uptake of home‐based HIV testing. The application was developed to address the most important perceived barriers for the uptake of HCT such as psychosocial factors, or existing norms and encourage to take immediate action towards HCT [38, 39]. However, unlike financial incentives which bring immediate and tangible benefit, overcoming psychosocial barriers through improving intrinsic motivation can be an iterative and slow process. Since HCT was offered right after the participants had listened to EPIC‐HIV, they might have not yet been ready to make immediate action towards HCT. Nevertheless, in the post‐intervention satisfaction surveys, 96% of participants who received EPIC‐HIV 1 found EPIC‐HIV 1 acceptable and motivated them to test, and almost everyone reported being empowered with the information from the app [40, 41], suggesting that engagement with EPIC‐HIV 1 would potentially lead to better uptake of home‐based HIV testing or linkage to care in the future.

The overall population coverage of HIV testing during this single round of testing was relatively low at 21% (with 32% of men away from home at the time of the fieldworker visits and 33% choosing not to participate in the annual population‐based HIV testing), although over several rounds of testing, both the cumulative contact rate and the level of HIV testing would increase. For example the consent rate to at least one HIV test reached over 75% between 2005 and 2016 [31], and approximately half of the eligible individuals consented at least twice within five years of becoming eligible [42]. Nevertheless, consistently reaching men for HIV testing and treatment remains a persistent challenge in this population and across multiple contexts [14, 22, 43]. A recent systematic review showed that acceptability of home‐based HIV testing greatly varies from 58.1% to 99.8% but with the high pooled proportion of 83.3% throughout sub‐Saharan Africa [44]. In the area neighbouring communities, where the ANRS TasP 12249 Trial was conducted, the uptake of home‐based HIV testing was also high at 82.9% [12]. When we limited our analysis to those who were present at home and offered the testing, the overall uptake of HIV testing was around 65%, and significantly higher in the intervention arms than in the SoC arm. Thus, it might require more than one visit to offer HIV testing and intervention in order to have a substantial impact at a population level.

Another challenge for delivering home‐based HIV testing is frequent short‐term mobility and long‐term migration. In the study area, more than 20% of the residents migrate at least once in a given year, putting them at a higher risk of HIV acquisition and lack of access to HIV testing, prevention, or treatment services [45]. Other innovative approaches have been shown effective to increase the uptake of HCT such as provision of HIV self‐testing kits during delivery of home‐based HIV testing services [46], at the community [47], or through female partners attending antenatal care clinics [19] as well as finding men and offering tests at convenient locations (e.g. workplace) or via mobile testing [48, 49]. Financial incentives coupled with innovative approaches to reach men and offer HIV testing at different settings or improve access to self‐testing kits may further improve the uptake of HIV testing.

The trial will further quantify the effect of financial micro‐incentives and EPIC‐HIV applications on enhancing linkage to care within three months to constitute the second primary outcome of the trial in late 2020. We will also quantify the long‐term effects on HIV‐related mortality among men and HIV incidence among young women by 2022 [34]. The trial has also collected detailed information on resources used to implement and deliver the interventions using micro‐costing and a time‐and‐motion approach and will quantify the cost‐effectiveness of financial micro‐incentives and EPIC‐HIV application to improve HIV testing uptake and linkage to care. A study in Tanzania showed that willingness‐to‐accept estimates for financially incentivized universal HIV testing among adults were $1.3‐$6.4 and considered highly cost‐effective [50], but there is very limited evidence for cost‐effectiveness of both financial incentives and behavioural intervention in the general population using the data from randomized clinical trials. Our study findings will provide important insights into the efficacy, cost‐effectiveness and sustainability of once‐off micro‐incentives and decision support application to reach men and improve long‐term population health outcomes in hyperendemic HIV settings.

The study has several important strengths. The study was the first of its kind and was delivered as a community‐randomized clinical trial among over 13,800 men in 45 communities nested within one of the world’s largest ongoing population‐based HIV cohorts. This allowed the unbiased measuring the effect of the interventions where home‐based HIV testing at households was a standard of care, controlling for potential contextual factors and differences across the communities, and provided sufficient power to detect the effects of interventions. In some randomized clinical trials, treatment non‐adherence or crossover among randomized groups may cause treatment contamination, thus a statistical approach like instrumental variables would be beneficial to adjust for treatment contamination [51]. In our study, however, every participant was offered and received interventions as randomized (i.e. no contamination of randomized interventions), further validating our intention‐to‐treat analysis. However, the study is not without limitations. In particular, men who did not participate in the annual HIV testing survey were not eligible to receive interventions in the study, thus ultimately limiting the potential population coverage of the interventions.

## 5 CONCLUSIONS

In summary, we found that a small, once‐off, financial incentive significantly improved the uptake of home‐based HIV testing by more than 50% among men in this large community‐randomized clinical trial. In contrast, the counselling and testing application which was designed to serve as an intrinsic motivator for HIV testing did not increase the uptake of home‐based testing. Financial micro‐incentives could be considered as an effective strategy to encourage the uptake of home‐based HIV testing, especially among the unreached population with a lower uptake of HCT.

## COMPETING INTERESTS

All co‐authors declare no conflict of interest.

## AUTHORS’ CONTRIBUTIONS

FT and TB are the principal investigators and developed the study design and protocol in collaboration with MS, SW, NM, JS, HMY, BS and AD. H‐YK & FT analysed the data and wrote the first draft of the manuscript. PM, AB, AZ, OA, TM, TZ, SW, JS and MS were involved in the development of EPIC‐HIV. All authors read and approved the final manuscript.

## ABBREVIATIONS

AHRI, Africa Health Research Institute; CONSORT, Consolidated Standards of Reporting Trials; EPIC‐HIV, Empowering People through Informed Choices for HIV; HCT, HIV Counseling and Testing; HIV, Human Immunodeficiency Virus; HITS, Home‐Based Intervention to Test and Start.

## Supporting information


**Text S1.** CONSORT 2010 checklist of information to include when reporting a randomised trial

**Figure S1.** Modified CONSORT flow diagram. Flow diagram shows the flow of communities and individuals through each stage of the cluster randomized controlled trial by intervention arms. EPIC, Empowering People through Informed Choices for HIV.

**Table S1.** Characteristics by participation status in the annual population‐based HIV testing in 2018 among all 13,838 eligible men
