# Supplementary material for: Home‐Based Intervention to Test and Start (HITS): a community‐randomized controlled trial to increase HIV testing uptake among men in rural South Africa
Source: J Int AIDS Soc. 2021 Feb 15;24(2):e25665. doi: 10.1002/jia2.25665 (PMC7883477; doi:10.1002/jia2.25665)
Supplement: Supplementary file 2 — Figure S1. Modified CONSORT flow diagram. Flow diagram shows the flow of communities and individuals through each stage of the cluster randomized controlled trial by intervention arms. EPIC, Empowering People through Informed Choices for HIV. [file JIA2-24-e25665-s002.pdf]

Home-Based Intervention to Test and Start (HITS): A community-randomized controlled trial to increase HIV testing uptake among men in rural South Africa

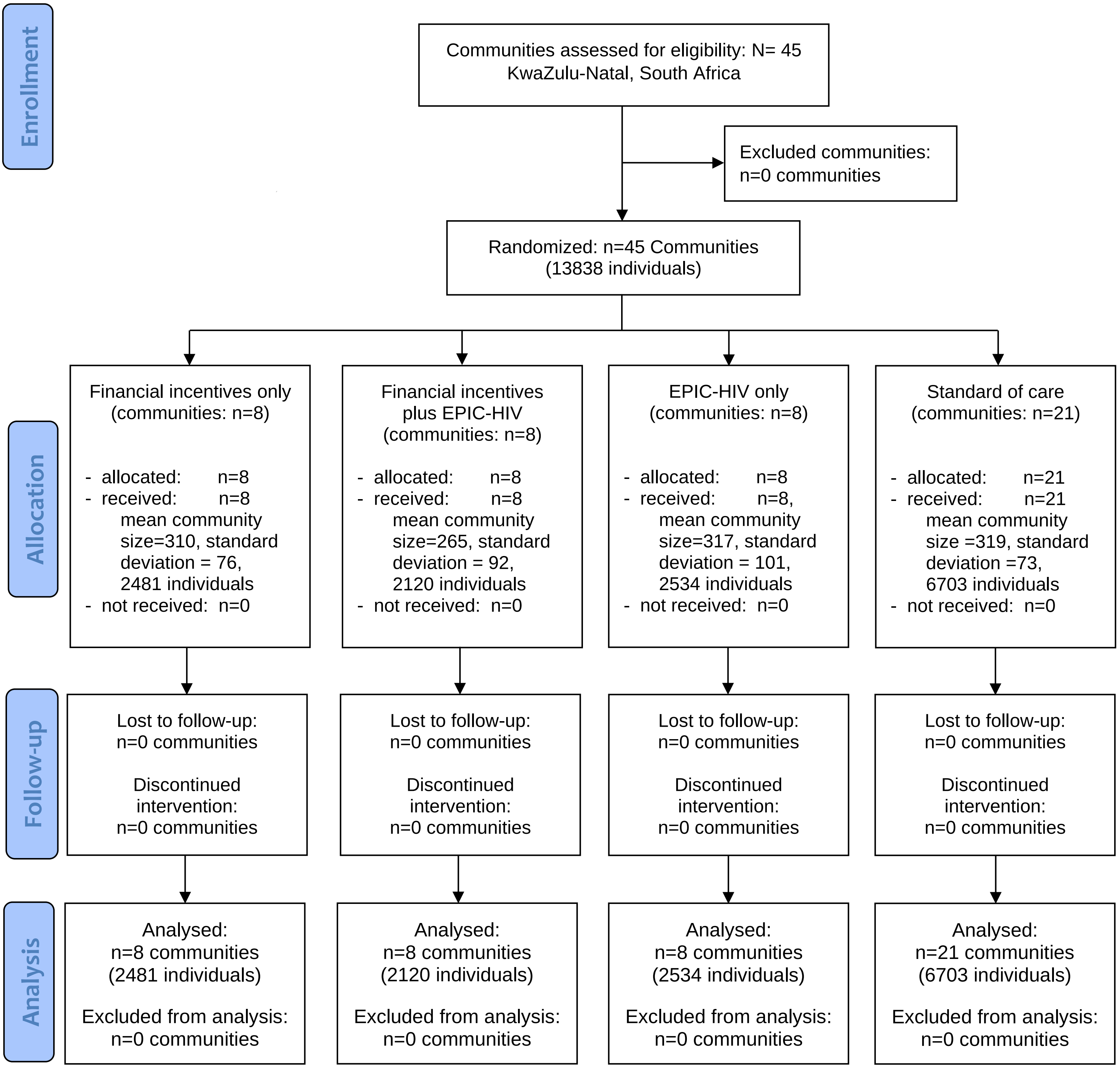


**Figure S1. Modified CONSORT flow diagram.** Flow diagram shows the flow of communities and individuals through each stage of the cluster randomized controlled trial by intervention arms. Abbreviation: EPIC, Empowering People through Informed Choices for HIV.
