# Supplementary material for: Home‐Based Intervention to Test and Start (HITS): a community‐randomized controlled trial to increase HIV testing uptake among men in rural South Africa
Source: J Int AIDS Soc. 2021 Feb 15;24(2):e25665. doi: 10.1002/jia2.25665 (PMC7883477; doi:10.1002/jia2.25665)
Supplement: Supplementary file 3 — Table S1. Characteristics by participation status in the annual population‐based HIV testing in 2018 among all 13,838 eligible men [file JIA2-24-e25665-s003.docx]

| **Table S1. Characteristics by participation status in the annual population-based HIV testing in 2018** **among all 13,838 eligible men** | | | | | |
| --- | --- | --- | --- | --- | --- |
| **Variable** | **(1) Never contacted** | **(2) Declined** | **(3) Consented** | **p-value** | **p-value** |
|  | n=4,444 | n=4,522 | n=4,872 | (1) vs. (3) | (2) vs. (3) |
| **Individual HIV testing history in the surveillance** |  |  |  |  |  |
| Time since last HIV test in the HIV surveillance (years), median (IQR) | 2.7 (1.0, 7.0) | 2.5 (1.0, 6.4) | 1.9 (1.0, 3.1) | <0.001 | <0.001 |
| Participation in the HIV surveillance when offered in the past (%), mean ± SD | 53.0 ± 38.2 | 50.4 ± 38.9 | 67.2 ± 34.3 | <0.001 | <0.001 |
| **Age (years), % (n)** |  |  |  |  |  |
| 15-25 | 33.6 (1517) | 32.8 (3600) | 43.5 (2120) | <0.001 | <0.001 |
| 25-35 | 24.1 (1088) | 25.0 (2743) | 18.4 (894) |  |  |
| 35-45 | 16.9 (764) | 17.8 (1948) | 11.5 (560) |  |  |
| 45-55 | 10.1 (456) | 11.1 (1218) | 8.6 (419) |  |  |
| ≥55 | 15.4 (696) | 13.3 (1461) | 18.0 (879) |  |  |
| **Marital Status, % (n)** |  |  |  |  |  |
| Never married | 24.9 (1127) | 25.8 (1146) | 28.9 (1407) | <0.001 | <0.001 |
| Married | 17.2 (777) | 12.7 (563) | 13.7 (668) |  |  |
| Informal Union | 1.9 (87) | 1.1 (48) | 2.5 (122) |  |  |
| Separated/Divorced/Widowed | 38.7 (1750) | 42.1 (1873) | 35.5 (1727) |  |  |
| Don't know/Missing | 17.3 (781) | 18.3 (814) | 19.5 (948) |  |  |
| **Education, % (n)** |  |  |  |  |  |
| No formal education | 20.3 (919) | 18.7 (833) | 26.5 (1291) | <0.001 | <0.001 |
| Primary (grade 1-7) | 5.3 (241) | 4.1 (180) | 6.9 (335) |  |  |
| Secondary+ (≥ grade 8) | 62.5 (2826) | 55.3 (2458) | 57.0 (2779) |  |  |
| Don’t know/Missing | 11.9 (536) | 21.9 (973) | 9.6 (467) |  |  |
| **Area of residency, % (n)** |  |  |  |  |  |
| Rural | 38.0 (1719) | 32.6 (1448) | 32.7 (1595) | <0.001 | 0.007 |
| Peri-urban | 52.9 (2391) | 59.9 (2660) | 61.3 (2988) |  |  |
| Urban | 9.1 (412) | 7.6 (336) | 5.9 (289) |  |  |
